# Editorial: Emerging infections in children

**DOI:** 10.3389/fped.2023.1168697

**Published:** 2023-03-10

**Authors:** Cecilia Perret

**Affiliations:** Department of Pediatric Infectious Diseases and Immunology, School of Medicine, Pontificia Universidad Católica de Chile, Santiago, Chile

**Keywords:** emerging infections, emergent viruses, children, antimicrobial resistance, pneumococcal meningitis, arboviruses emergence

**Editorial on the Research Topic** Emerging infections in children

Emerging infectious diseases, defined as infections that have newly appeared in a population or have existed but are rapidly increasing in incidence or geographic range, have undoubtedly gained more relevance since the COVID-19 pandemic. When we talk about emerging infections, we are referring not only to new pathogens but also to those who are introduced to new territories, those who develop resistance to common antimicrobials and those considered once under control, but whose incidence start to rise again. Most of the new pathogens infecting humans are viruses that mainly affect the respiratory tract, such as, SARS, SARS-CoV-2, avian influenza, and MERS. The behavior of these infections varies within the pediatric population and with respect to adults (1). As an example of pathogens introduced into new geographic sites are the arboviruses chikungunya and Zika, which were introduced into the Caribbean and the Americas in 2013 and 2014 respectively, causing a major epidemic in the continent (2), and affecting children and adults. Bacteria considered as emergent are those that change their resistance patterns, particularly Gram-negative bacilli. During the last few years, a rapid and significant increase in infections caused by bacteria producing expanded spectrum Beta lactamases (ESBL), AmpC and/or resistant to carbapenems are a concern (3) due to their significant morbidity and mortality. Finally, the step back in the control of immunopreventable diseases, such as invasive pneumococcal infections, due to the appearance of serotypes non included in vaccines, or due to changes in their virulence, is another way of presentation of emerging infections (4).

Usually, the knowledge about epidemiology, clinical manifestations, and therapeutic tools for these emerging infections in children is late and to a lesser extent than for adults. Hence the importance of the articles included in this collection, that touch aspects of emerging infections from the perspective of children, contributing to increase the information applicable for the pediatric population. Thus, with respect to SARS-CoV-2, the article by Pérez et al., contributes to narrowing the gap between adult and children information regarding to COVID-19, analyzing the disease in the population of children under 2 years of age. The authors note that it is a mild to moderate disease, which does not require hospitalization in the vast majority of children, that fever is the most frequent form of presentation and in children under 6 months, conjunctivitis appears as a characteristic symptom. After the onset of the Chikungunya epidemic in the Americas, during the great epidemic of 2014–2015, little was known about the impact and clinical manifestations in children. Damião- Gomes el al., describes the manifestations of CHIK in a pediatric population of Rio de Janeiro in Brazil, being very similar to that of adults, a mild to moderate disease, which presented with fever in 90%, arthralgias in 76.5%, and 45.1% with elevation of liver enzymes among the confirmed cases. This is a very interesting finding as many other articles show that children had less frequency of arthralgia compared to adults with less liver compromise (5). Resistance to gram-negative due to the presence of bacteria producing expanded spectrum beta-lactamases (ESBL) is a growing problem that also affects the pediatric population, increasing morbidity and mortality. Treatment of ESBL bacterial infections places carbapenems as the drugs of choice against invasive ESBL producing bacteria (6). The article by Zhao et al., presents a meta-analysis on the use of ertapenem in children, a carbapenem, demonstrating that there are no differences in the efficacy and safety compared to beta-lactams in children, and can be considered as a tool for the management of bacteria resistant to beta-lactams, producers of ESBL, considering its convenient PK and PD, particularly for intra-abdominal infections and UTIs (7, 8). The introduction of pneumococcal conjugate vaccines (PCV) against *Streptococcus pneumoniae*, the main causative agent of bacterial meningitis in children, was a significant milestone in reducing morbidity and mortality in the pediatric population. Several years after the incorporation of different vaccines that cover a certain number of pneumococcal serotypes, this powerful effect seems to be changing. Farfán-Albarracín et al., describes the increase in the incidence of pneumococcal meningitis in Colombia after the introduction of 10 valent PCV, due to serotypes not included in the vaccine and with higher antibiotic resistance. This phenomenon has been also described elsewhere such as Europe and the USA (9) being a global problem. Strict surveillance of invasive pneumococcal disease and the appearance of new serotypes taking in account their antimicrobial resistance and virulence, is very necessary to be able to control this phenomenon in a more effective way.

The impact of emerging infections, in all its dimensions, cannot be extrapolated to children from the adults. Its epidemiology, clinical manifestations and treatment are particular for the pediatric population. The contribution to the knowledge made by the authors participating in this collection is appreciated.
